# Characterization of the diffusion coefficient of blood

**DOI:** 10.1002/mrm.26919

**Published:** 2017-09-23

**Authors:** Carsten Funck, Frederik Bernd Laun, Andreas Wetscherek

**Affiliations:** ^1^ Medical Physics in Radiology, German Cancer Research Center (DKFZ) Heidelberg Germany; ^2^ Institute of Radiology University Hospital Erlangen Erlangen Germany; ^3^ Joint Department of Physics at The Institute of Cancer Research and The Royal Marsden NHS Foundation Trust London United Kingdom

**Keywords:** MRI, diffusion, IVIM, flow compensation, blood, perfusion

## Abstract

**Purpose:**

To characterize the diffusion coefficient of human blood for accurate results in intravoxel incoherent motion imaging.

**Methods:**

Diffusion‐weighted MRI of blood samples from 10 healthy volunteers was acquired with a single‐shot echo‐planar‐imaging sequence at body temperature. Effects of gradient profile (monopolar or flow‐compensated), diffusion time (40–100 ms), and echo time (60–200 ms) were investigated.

**Results:**

Although measured apparent diffusion coefficients of blood were larger for flow‐compensated than for monopolar gradients, no dependence of the apparent diffusion coefficient on the diffusion time was found. Large differences between individual samples were observed, with results ranging from 1.26 to 1.66 µm^2^/ms for flow‐compensated and 0.94 to 1.52 µm^2^/ms for monopolar gradients. Statistical analysis indicates correlations of the flow‐compensated apparent diffusion coefficient with hematocrit (*P* = 0.007) and hemoglobin (*P* = 0.017), but not with mean corpuscular volume (*P* = 0.64). Results of Monte‐Carlo simulations support the experimental observations.

**Conclusions:**

Measured blood apparent diffusion coefficient values depend on hematocrit/hemoglobin concentration and applied gradient profile due to non‐Gaussian diffusion. Because in vivo measurement is delicate, an estimation based on blood count results could be an alternative. For intravoxel incoherent motion modeling, the use of a blood self‐diffusion constant D_b_ = 1.54 ± 0.12 µm^2^/ms for flow‐compensated and D_b_ = 1.30 ± 0.18 µm^2^/ms for monopolar encoding is suggested. Magn Reson Med 79:2752–2758, 2018. © 2017 The Authors Magnetic Resonance in Medicine published by Wiley Periodicals, Inc. on behalf of International Society for Magnetic Resonance in Medicine. This is an open access article under the terms of the Creative Commons Attribution License, which permits use, distribution and reproduction in any medium, provided the original work is properly cited.

## INTRODUCTION

“Blood is a juice of very special kind” 1 applies no less in the context of its MRI properties. One reason for its complex behavior is that its two main constituents, blood plasma (55%) and red blood cells (45%) 2, undergo exchange of water molecules 3, 4. Not only have blood‐relaxation times 5, 6 and their dependence on oxygenation level been studied 7, 8, 9, but also the effects of diffusion were modeled and measured by Stanisz and Li 10, 11. They took into account restricted diffusion in the red blood cells (RBCs), hindered diffusion in the plasma, and exchange between those compartments in a pulsed field gradient multi‐spin‐echo sequence 12 using short gradient pulses (G_max_ = 6500 mT/m). The use of short pulsed gradients enables the measurement of the average displacement propagator 13, and therefore can yield a good estimate of diffusion properties; however, on a typical clinical scanner (G_max_ = 40 mT/m), diffusion gradients are elongated to achieve a sufficiently strong weighting. In this case, application of the approach by Stanisz and Li 10, 11 and derivation of an analytical expression is not straightforward.

Apart from staging hematomas 14, blood diffusion properties play an important role in intravoxel incoherent motion (IVIM) imaging 15, which allows one to separate the effects of blood motion and tissue water diffusion 16. In this framework, the diffusion‐weighted signal attenuation caused by blood flow is modeled via a pseudo‐diffusion coefficient D*, which is much larger than the self‐diffusion coefficient D_b_ of blood. Although D_b_ is therefore negligible in typical IVIM experiments, this is not the case if flow‐compensated (FC) diffusion gradients are used, which was first proposed by Ahn 17 and Maki 18. In a recent publication, the utility of using both monopolar (MP) and FC diffusion gradients for IVIM measurements in liver and pancreas was shown, which has the potential to measure characteristic length and velocity in vascular networks 19. The method presented here, however, used a fixed value of D_b_ = 1.6 µm^2^/ms, which was an estimate based on 10, 11.

In this work, the apparent diffusion coefficient (ADC) of blood is characterized using a diffusion‐weighted sequence on a clinical scanner. The multicompartment nature of blood is considered by varying the echo time (TE), diffusion time T, and diffusion gradient profile (FC or MP), and Monte Carlo simulations of blood diffusion are performed. Although the initial intent of this study was to provide an accurate estimate of D_b_ for FC IVIM 19, its results might also be relevant in the case of cardiac diffusion MRI 20, 21, in which FC gradients are used, and add to the ongoing debate on the value of diffusion‐weighted MRI in the assessment of intracranial hemorrhage 22.

## METHODS

**Figure 1:**
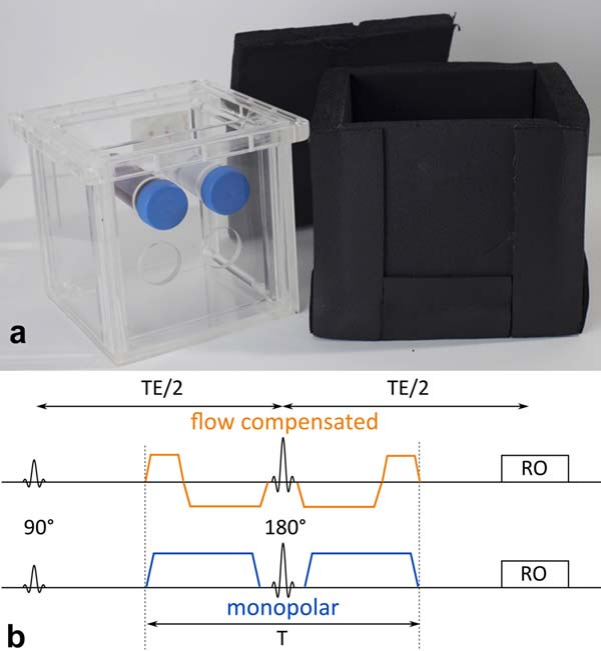
**a**: Water tank with one blood sample and one water tube as a reference is placed inside foam insulation (right side). Sample tubes are manually rotatable around their long axis without the need to move the phantom between measurement steps. **b**: Sequence timing of the used diffusion MRI sequence with FC and MP gradient profiles. Diffusion time T can be varied independently of TE.

A phantom assembly was built holding rotatable sample tubes in a plastic frame within a water tank (Fig. 1a). Foam isolation was used to reduce cooling to less than 1°C during the 30‐min MRI protocol, which was controlled using an analog thermometer. Blood was drawn from 10 consenting healthy volunteers (5 male, 5 female) via venipuncture of the forearm. Heparin was added (19 IU/mL), and blood was stored in a sample tube (60 mL) in a water bath at 37.5°C for up to an hour before it was measured. As depicted in Figure 1a, we always measured one blood sample simultaneously with one water‐reference tube. For each blood sample, a blood count was obtained. The measurement protocol was divided into steps of less than 3‐min duration, such that sample tubes could be rotated in between acquisitions to prevent sedimentation 8.

The phantom was placed inside a 12‐channel head coil with sample tubes orientated parallel to B_0_ = 1.5 T (Magnetom Symphony, Siemens Healthcare, Erlangen, Germany). An in‐house‐developed diffusion‐weighted single‐shot spin‐echo echo‐planar‐imaging sequence was used, which allowed for setting the duration T of the diffusion‐weighting block independently of TE 19. Both MP and FC diffusion gradients were implemented (Fig. 1b). The imaging parameters were as follows: field of view: 250 × 195 mm^2^, nominal in‐plane resolution: 2.5 × 2.5 mm^2^, imaging matrix 100 × 78, slice thickness/gap: 5/2.5 mm, readout bandwidth: 2000 Hz/Px, repetition time = 2.5 s, partial Fourier: 0.75, Grappa 2. For each combination of T and TE, an unweighted scan was added before and after the diffusion‐weighted scans, for which b‐values 50 and 400 s/mm^2^ were applied along the six directions (1,1,0), (1,0,1), (0,1,1), (−1,−1,0), (−1,0,−1), (0,−1,−1). First, T was varied between 40 and 100 ms (FC: 70 to 100 ms) in steps of 10 ms with fixed TE = 120 ms (T‐dependence). Second, TE was varied between 60 and 200 ms with diffusion times fixed to the minimum time required to achieve b = 400 s/mm^2^, which was T_min_ = 40 ms for MP and T_min_ = 70 ms for FC (TE‐dependence). Diffusion gradients were applied symmetrically around a refocusing pulse to minimize artifacts related to concomitant fields. For details on the implementation see 19. As highlighted by Stoeck et al, the gradient profile is second‐order motion‐compensated 23. An alternative implementation was demonstrated by Ahlgren et al. 24.

The ADC calculation was based on regions of interest and using all three b‐values. Mean signal intensities were averaged over all diffusion‐gradient directions and slices, as no dependence on the slice position was observed. A noise correction of the measured signal *S*_*m*_ according to $S_{c}=\sqrt{S_{m}^{2}-6\sigma^{2}}$ was performed, where σ is the average background noise of one of the six individually accessible receiver channels measured in the image outside the phantom. The ADC measurements, in which $S_{c}$ was less than $\sqrt{60}\sigma$ in the b = 400 s/mm^2^ image (equivalent to signal‐to‐noise ratio (SNR) < $\sqrt{10}$ ≈ 3.2) were excluded to avoid underestimation of the ADC. The factors 6 and $\sqrt{60}$ were derived for the sum‐of‐squares reconstruction based on the finding that the lower coil elements yield approximately half of the signal of the upper coil elements within the sample tubes, and assuming that each coil element can be corrected according to $\sqrt{S^{2}-\sigma^{2}}$ 25 (for details see Supporting Text 1).

The Pearson product moment coefficient *r* was calculated for each combination of ADC with hematocrit (HCT), mean corpuscular volume (MCV), and hemoglobin concentration (HGB). Linear regression was performed in IGOR pro (WaveMetrics Inc, Portland, OR), if the null hypothesis of uncorrelated parameters was rejected at a significance level of 5% (|*r*| > *r*_crit_). A paired two‐sided t‐test was used to compare MP and FC ADC values.

Monte Carlo simulations of blood diffusion were performed in MATLAB (R2015b, the MathWorks Inc, Natick, MA). Source code is available from https://github.com/awetscherek/bloodMC (SHA1: xxx). The RBC shape was modeled by oblate cylinders of 8.00 µm diameter and height of 1.75 µm, which were scaled to match the sample MCV. The RBC was placed centered in a cuboid unit cell, sized such that HCT equaled RBC volume fraction. The resulting surface‐to‐volume ratio is 1.64 µm^‐1^, which is close to the typical surface‐to‐volume ratio of 1.5 µm^‐1^ for red blood cells reported by Linderkamp 26. Diffusion in plasma was governed by *D*_*p*_ = 2.75·10^‐3^ mm^2^/s, as reported for 37°C 10, and by *D*_*e*_ = 1.00 · 10^‐3^ mm^2^/s in erythrocytes. With free‐water concentrations of *c*_*p*_ = 0.95 and *c*_*e*_ = 0.70, the fraction of blood water contained in RBCs is given by f = *c*_*e*_ · HCT/[*c*_*e*_ · HCT + *c*_*p*_ · (1 ‐ HCT)] 6. The probability for transmission from compartment *i* is modeled as *P* = 2 *d*_*s*_ *κ*_*i*_/*D*_*i*_ following 27, where *d*_*s*_ is the distance to the membrane. We chose a pre‐exchange lifetime of *τ*_*e*_ = 12 ms based on the range reported in literature for water molecules in RBCs 28, yielding the membrane permeability *κ*_*e*_ = (*V*/*S*)/*τ*_*e*_ with the surface‐to‐volume ratio of the RBCs. For stable concentrations, *κ*_*e*_ = *κ*_*p*_ · (*c*_*p*_/*c*_*e*_) follows.

A total of 300,000 particle trajectories x(t) were simulated for a duration of TE = 120 ms, and trajectory center of mass location $x_{\text{cm},k}=\frac{1}{\Delta t_{k}}\int_{t_{k-1}}^{t_{k}}x\left(t\right)dt$ was saved for each of the *K* time intervals Δ*t*_*k*_ = *t*_*k*_ − *t*_*k*‐1_ of constant diffusion gradient. Gradients are switched on at *t*_*0*_ and off at *t*_*K*_ with gradient sign switching at *t*_*k*_ with 0 < *k* < *K*. For the sequence in use, those time points are given by *t*_*k*_ = TE/2 + (*k*‐1)·T/2 for the MP (*k* ∊ 0,1,2) and *t*_*k*_ = TE/2 + T/4·sgn(k‐2)· ${\sqrt{2}}^{\left|k-2\right|}$ for the FC profile (*k* ∊ 0,1,2,3,4), as described in 19. The effective gradient profiles used in the simulations are visualized in Supporting Figures S1a (MP) and S1b (FC). Step size was chosen as 1% of the shortest dimension (distance between neighboring RBCs). For example, in the case of HCT = 43% and MCV = 87.7 fL, this results in a step size of 8.56 nm, resulting in a total of 2.7 · 10^7^ steps for TE = 120 ms. The runtime ranged from 5.5 to 12.5 h on an Intel Xeon E5‐1660 v3 with 128 GB memory. Distributions of accumulated phase φ were obtained by weighting ***x***_cm_ along 10000 diffusion gradient directions **g** for isotropic weighting, where the magnitude of **g** corresponded to b = 400 s/mm^2^:

(1) $$\varphi =\varphi \prime \sqrt{b}=\gamma g\cdot \overset{K}{\underset{k=1}{\sum }}\Delta t_{k}x_{cm,k}{\left(-1\right)}^{k}$$

This connection between φ and $x_{\text{cm},k}\;$ goes back to 29. Recognizing that φ is proportional to |**g**|, and thus $\sqrt{b}$, allows one to introduce a normalized phase φ′, similar to the concept used in 19. From the results of the Monte Carlo simulations, the distribution of normalized phases φ′ can be obtained for each combination of gradient profile and diffusion time. From those one can calculate ${\text{ADC}}_{b}=-\frac{1}{b}\ln\left|〈e^{i\varphi^{\prime }\sqrt{b}}〉\right|$, which is the ADC value that one would obtain from one measurement at b and one without diffusion weighting (b = 0).We also calculated excess diffusional kurtosis 30 $K_{\text{app}}=〈{\varphi^{\prime }}^{4}〉/{\left(〈{\varphi^{\prime }}^{2}〉\right)}^{2}-3$ and the limit of ADC_b_ for small b‐values, ${\text{ADC}}_{0}:=\lim_{b\to 0}{\text{ADC}}_{b}=〈{\varphi^{\prime }}^{2}〉/2,\;$ which is equivalent to the Gaussian phase approximation as shown elsewhere 19. According to Equation [36] in 30, those properties are linked as follows:

(2) $$ADC_{b}=-\frac{1}{b}\ln\frac{S\left(b\right)}{S\left(0\right)}=ADC_{0}-\frac{b}{6}ADC_{0}^{2}K_{app}+O\left(b^{2}\right)$$

## RESULTS

**Figure 2:**
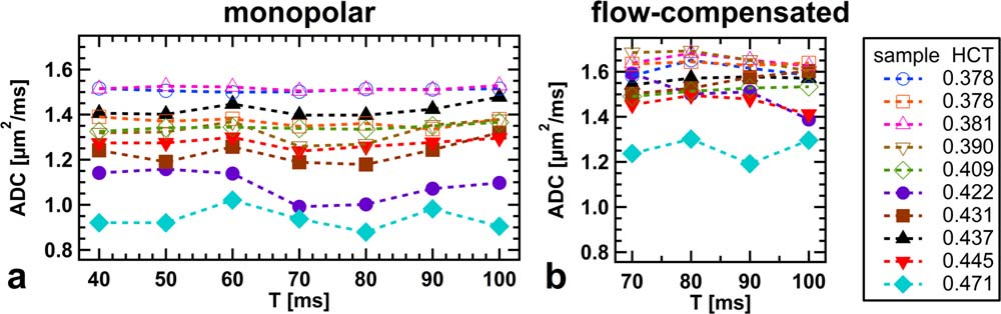
No characteristic dependence of measured ADC on diffusion time T is visible in the blood samples. Blood ADCs are larger for FC gradients (**b**) than for MP gradients (**a**). Samples with lower HCT exhibit a tendency toward higher ADC. The ADCs concurrently measured in water samples are provided as Supporting Figure S1.

Measured ADCs are plotted against diffusion time T in Figure 2 for blood samples (Figs. 2a and 2b). Data from simultaneously acquired water reference tubes are provided as Supporting Figures S1c and S1d. Monopolar ADCs are displayed on the left (Fig. 2a), whereas FC ADCs are displayed on the right (Fig. 2b). Different markers and colors are used to distinguish blood samples (HCT is displayed in the legend), where unfilled markers depict samples from female volunteers. Errors for individual ADC measurements based on the analysis of 95% confidence intervals were in the range of ±(0.03–0.07) µm^2^/ms for MP gradients and in the range of ±(0.06–0.11) µm^2^/ms for FC measurements, in which samples with faster T_2_ relaxation exhibit lower SNR, resulting in higher variance.

Although ADCs measured in the water reference agree within error margins across all measurements and are in accordance with literature 31, blood ADCs from different samples differ strongly for both MP and FC diffusion gradients. Because the uncertainty in temperature adjustment is only of the order of 1°C, ADC differences between blood samples must be attributed to different blood composition. Flow‐compensated diffusion weighting yields higher ADC values compared with MP gradients for blood (*P* < 10^‐14^), whereas ADCs measured in the water reference are independent of the gradient profile (*P* = 0.083). Considering all measurements, no characteristic dependence of the ADC on T is apparent.

### Hematocrit, MCV, and HGB Dependence

**Figure 3:**
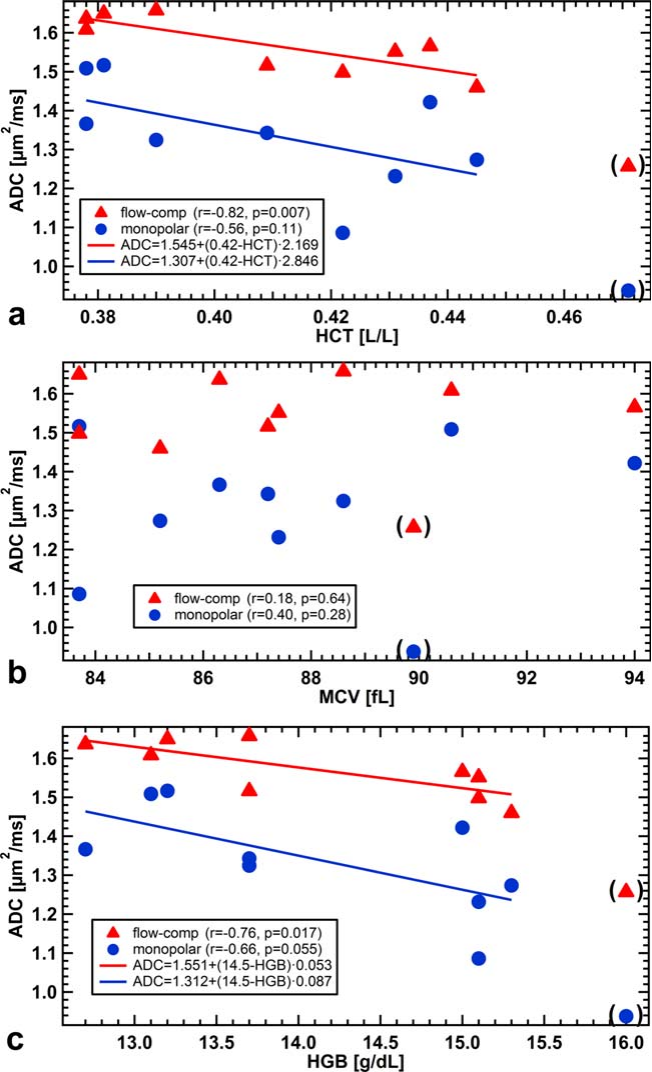
ADCs averaged over all diffusion times versus hematocrit (**a**), mean cell volume (**b**), and hemoglobin concentration (**c**) for FC and MP diffusion gradients. Linear regression was performed, where |r| > r_crit_ = 0.666. Although ADCs decrease for increasing HCT and HGB (significant only for FC gradients), no trend is apparent for MCV. Flow‐compensated diffusion gradients yield larger ADCs. Data points in parentheses suffered from very low SNR and were excluded from the analysis (see also Fig. 4).

For each sample, ADCs averaged over all diffusion times were plotted in Figure 3 against HCT, MCV, and HGB. Results from measurements with FC and MP diffusion encoding are depicted by different markers and colors. Because statistical analysis indicated a correlation between ADC and HCT (FC: r = −0.82, *P* = 0.007; MP: r = −0.56, *P* = 0.11), linear regression was performed yielding ADC_FC_[µm^2^/ms] = 1.545 + (0.42 − HCT) · 2.169 and ADC_MP_[µm^2^/ms] = 1.307 + (0.42 − HCT) · 2.846. No ADC dependence on MCV becomes apparent (FC: r = 0.18, *P* = 0.64 and MP: r = 0.40, *P* = 0.28), whereas ADC versus HGB shows a trend similar to the relation between ADC and HCT (FC: r = −0.76, *P* = 0.017 and MP: r = −0.66, *P* = 0.055): ADC_FC_[µm^2^/ms] = 1.551 + (14.5 – HGB[g/dL]) · 0.053 and ADC_MP_[µm^2^/ms] = 1.312 + (14.5 ‐ HGB[g/dL]) · 0.087.

**Figure 4:**
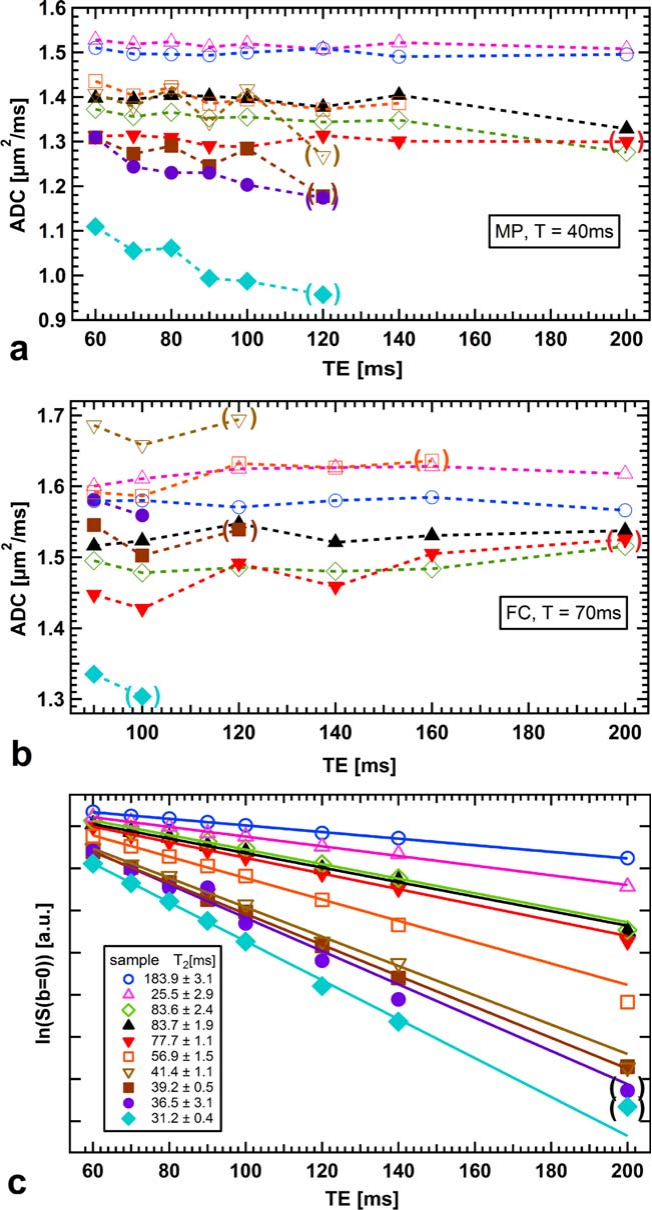
Blood ADC versus echo time for MP (**a**) and FC (**b**) diffusion weighting. Sample markers and colors equal those in Figure 2. Data points in parenthesis are based on measurements with low SNR ( < 5). Although ADCs do not exhibit clear TE dependence for samples with T_2_ > 50 ms, ADCs decrease with TE for T_2_ < 40 ms in the MP case. **c**: Logarithmic unweighted signal intensity versus echo time. Mono‐exponential fits were used to estimate T_2_.

### Echo Time Dependence

Figure 4 shows the result of the second measurement series, for which the echo time was varied, whereas the diffusion time was fixed. Individual samples are marked as in Figure 2. Using the unweighted (b = 0 s/mm^2^) measurements from this series, T_2_ relaxation times were calculated (Fig. 4c). Because of the short T_2_ relaxation time of some samples, the SNR of the diffusion weighted image at longer TE is not sufficient to provide reliable ADC measurements for those samples. In Figures 4a and 4b, only data points in which the signal at b = 400 s/mm^2^ fulfilled the SNR criterion (SNR > 3.2) are displayed, and those with 3.2 < SNR < 5 are marked with parentheses. The two samples with the shortest T_2_ relaxation times were found to show a significant decrease (*P* = 0.014 and 0.028) of the ADC with TE (Fig. 4a), whereas samples with longer T_2_ relaxation time were not found to exhibit a particular TE dependence.

### Simulation

**Figure 5:**
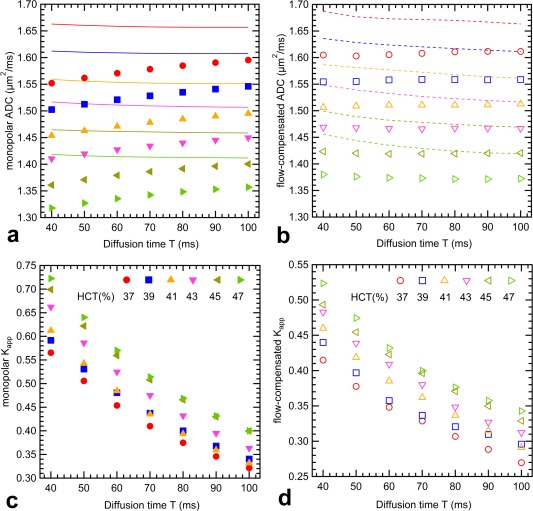
Simulation results for MP (**a, c**) and FC (**b, d**) gradients for different diffusion times and fixed TE = 120 ms. Hematocrit is the only differing parameter, and assignment of color is shown in (**c**) and (**d**). ADC_0_ is denoted by lines and does not show a T dependence in the MP case (**a**), whereas ADC_0_ decreases with T in the FC case (**b**). K_app_ > 0 indicates non‐Gaussian diffusion (**c, d**) and decreases with T, which is reflected in the T dependence of ADC_400_ (**a**). Effects balance out in the FC case (**b**).

Figure 5 shows the results of the simulations, which were performed for different HCT values over the HCT range present in our blood samples (denoted by different colors; see Figs. 5c and 5d). The MCV was set to the mean value of the experimental blood samples, which was 87.7 fL. ADC_0_ = 〈φ^2^/2〉/b (assuming Gaussian phase distribution) was calculated and shows no T dependence for MP gradients (continuous lines in Fig. 5a), whereas a decreasing ADC_0_ is found for FC gradients (dashed lines in Fig. 5b), which approaches the values of Figure 5a for longer diffusion times. Excess diffusional kurtosis is a measure of the deviation of the phase distribution from a Gaussian function, and decreases for both gradient profiles at larger T (Figs. 5c and 5d). It is directly related to the measured ADC_b_ according to Equation [2], explaining the smaller values of ADC_400_ (markers in Figs. 5a and 5b), when compared with ADC_0_.

The simulation confirms the experimental findings (Fig. 2) that the measured ADC exhibits a strong dependence on HCT. It should be noted, however, that the range of ADC_400_ values in the simulation (Figs. 5a and 5b) is smaller (1.33–1.58 µm^2^/ms) than in the experiment (0.9–1.65 µm^2^/ms), which presumably reflects that the simplified model is only a rough approximation of the real situation.

## DISCUSSION

Diffusion‐weighted imaging was performed on blood samples of 10 healthy volunteers using a phantom and measurement protocol devised to minimize the effects of sedimentation 8 and cooling. A water‐reference sample was acquired simultaneously, and the ADC measured in water did not exhibit any dependence on diffusion time, TE, or on the applied diffusion gradient profile, which ensured that applied b‐values were correct for the in‐house‐developed MRI sequence 19.

The finding that ADCs measured with FC gradients were higher than those obtained from MP gradients can be explained by two effects. First, in the concept of “effective diffusion time” T_eff_, the FC profile can be understood as three oscillations of the bipolar profile, resulting in T_eff,FC_ ≈ T_eff,MP_/3. A measurement with shorter T_eff_ is less sensitive to diffusion restriction by the RBC membrane and thus exhibits a higher ADC_0_ 32, which is confirmed by our simulations (Figs. 5a and 5b). The second effect is related to diffusional kurtosis, which causes a reduction of measured ADC_b_ compared with ADC_0_. Our simulations reveal that K_app_ is higher for MP gradients (Fig. 5c) than for FC gradients (Fig. 5d), further increasing the difference in ADC_400_ between FC and MP gradients (Figs. 5a and 5b). Similar results were found by Portnoy et al. 33, who speculated that the different kurtosis found for (non‐FC) pulsed‐gradient spin‐echo and (FC) oscillating‐gradient spin‐echo pulse sequences originated from the different gradient spectrum F(ω). The dependence of the kurtosis on T found in Figures 5c and 5d could be related to exchange between the compartments, which was found by Jensen and Helpern 34.

Although the used b‐value of 400 s/mm^2^ is rather low compared with the optimal b‐value of b = 1.278/ADC that one would choose in the case of a mono‐exponential decay 35, 36, the influence of the kurtosis term is not negligible; it can be understood from Figure 5b that the two T dependencies from restriction and exchange balance out for our choice of simulation parameters. The increase in ADC_400_ between T = 40 ms and T = 100 ms predicted by the simulations in Figure 5a is less than 0.05 µm^2^/ms, which is of the order of our measurement uncertainty, explaining why it was not revealed by our experimental data. A characterization with substantially increased SNR, such as at higher field strength, would be necessary to experimentally resolve a possible T dependence of the blood ADC. An experimental characterization of the diffusional kurtosis would have made our work more complete, but the necessary measurements at high b‐values with the used FC gradient profile could only be achieved with high‐performance gradient coils.

Although our experimental results would indicate that there is a correlation between HCT/HGB and blood ADC, it is only significant for FC gradients in this study. It is backed up by the simulations, which also show a strong dependence of ADC on HCT values, but the range of ADC values is not as large as in the experiment. In preparatory simulations, we found that this range and difference in ADC between MP and FC gradients depend heavily on the chosen membrane permeability. For the simulations presented here, we chose to stick to a literature value of τ_e_ = 12 ms based on 28, which was in better agreement with our experimental data than the range of τ_e_ = 6–9 ms reported in 37. Choosing an even longer intracellular pre‐exchange lifetime than τ_e_ = 12 ms for the simulations (corresponding to a lower membrane permeability) would have matched our experimental data better. This could be related to the approximation of the biconcave disc shape by a cylinder (changing the surface‐to‐volume ratio and thus membrane permeability *κ*_*e*_) or to membrane degradation after blood was taken. Despite frequent mixing, orientation of the erythrocytes in the static magnetic field might have played a role 38.

One limitation of our study is that blood oxygenation was not recorded. Blood oxygenation is well known to influence T_2_ 39, 40, 41, with lower oxygen saturation resulting in shorter relaxation times as a result of magnetic field gradients caused by susceptibility differences between RBCs and plasma 8. This is the basis for blood‐oxygenation‐level dependent (BOLD) contrast imaging 42. A further study measuring blood diffusion with monitoring of the blood oxygenation level would be needed to separate the effects of HCT/HGB and those originating from changes in blood oxygenation on the whole‐blood ADC. Relaxation time effects and additional gradients caused by susceptibility mismatch could be incorporated into the Monte‐Carlo simulations, which might affect the simulation results of the ADC dependence on T (Figs. 5a and 5b), but could also give insight into the dependence of the ADC on TE, which we experimentally observed in some samples (Figs. 4a and 4b).

Although we tried to exclude low SNR as a cause, the decline in ADC remains significant for the two samples with the fastest T_2_ relaxation when limiting our analysis to data with SNR greater than 5. A TE dependence of the blood ADC could only be explained by different relaxation times and diffusivities in plasma and RBCs. Although those conditions are fulfilled, the system is in the fast exchange limit for relaxation, thus exhibiting only a mono‐exponential T_2_ relaxation (Fig. 4c) and suppressing a TE dependence of the ADC. A possible explanation for the observed TE dependence in the samples with the fastest T_2_ relaxation could thus be membrane degradation and reduced exchange as a consequence of the presumably lower oxygen saturation. This theory, however, could only be proven in a study in which oxygen saturation of the blood samples was measured.

Another limitation is that blood composition differs between different types of blood vessels. Apart from changes in oxygen and carbon dioxide concentrations, hematocrit 43 and erythrocyte volume 44 differ between blood in veins and capillaries, which will affect the ADC. As the characteristic length scales obtained with FC IVIM 19 would indicate, the IVIM effect is caused primarily by blood flow in arterioles and venules, with the latter containing 4 times more blood volume 45. Assuming that blood constitution in veins and venules is similar, our results should be applicable for IVIM.

In conclusion, we found no dependence of the ADC on diffusion time T in the range of 40 to 100 ms. The ADCs measured with FC gradients were larger than those obtained with MP encoding. Large ADC differences between samples were found, correlating with HCT and HGB. For accuracy of FC IVIM measurements, we recommend the use of a blood ADC of 1.54 ± 0.12 µm^2^/ms for FC encoding and 1.30 ± 0.18 µm^2^/ms for MP encoding. If a blood count is available, we recommend the use of ADC_MP_[µm^2^/ms] = 1.307 + (0.42 − HCT) · 2.846 and ADC_FC_[µm^2^/ms] = 1.545 + (0.42 − HCT) · 2.169, respectively.

## Supporting information


**Text 1.** Derivation of factors for noise correction and low SNR data exclusion criterion.
**Fig. S1. a, b**: Effective diffusion‐weighting profiles used for the Monte Carlo simulations, which correspond to the diffusion sequence displayed in Figure 1b. To obtain a particular b‐value, the waveforms have to be scaled by the gradient amplitude g such that $b=a\gamma^{2}g^{2}T^{3}$ with a=1/12 for the MP profile (**a**) and $a=\sqrt{2}/8-1/6$ in the FC case (**b**). **c, d**: ADCs measured in a water‐reference tube simultaneously with the blood samples (same marker as the respective samples in Fig. 2). Water ADCs agree within 3% error across all measurements and with literature values.
