# Supplementary material for: Characterization of the diffusion coefficient of blood
Source: Magn Reson Med. 2017 Sep 23;79(5):2752–8. doi: 10.1002/mrm.26919 (PMC5836916; doi:10.1002/mrm.26919)
Supplement: Supplementary file 1 — Text 1. Derivation of factors for noise correction and low SNR data exclusion criterion. Fig. S1. a, b: Effective diffusion‐weighting profiles used for the Monte Carlo simulations, which correspond to the diffusion sequence displayed in Figure 1b. To obtain a particular b‐value, the waveforms have to be scaled by the gradient amplitude g such that b=aγ2g2T3 with a=1/12 for the MP profile (a) and a=2/8−1/6 in the FC case (b). c, d: ADCs measured in a water‐reference tube simultaneously with the blood samples (same marker as the respective samples in Fig. 2). Water ADCs agree within 3% error across all measurements and with literature values. [file MRM-79-2752-s001.pdf]

Supporting Information: Noise Correction and Data Exclusion Criterion

Basic considerations

A sum of squares coil combination was used for image reconstruction. We evaluated the coil sensitivities based on the individual coil images for our measurement setup. The sequence interface allowed only access to six combined coil channels. Those could be grouped into “upper” (covering anterior part of the head) and “lower” (posterior) channels. We measured, that the “upper channels” (index 1) yield approximately twice the signal of the “lower channels” (index 2), since the sample tubes were placed in the upper two phantom inserts (as depicted in FIG.1a in the manuscript).

We therefore can assume the following relation between the measured signals $S_{m,1}$ in an upper channel and $S_{m,2}$ in a lower channel (for our sample tube position): $S_{m,1}^2 = \left(2S_{m,2}\right)^2 = 4S_{m,2}^2$

Noise Correction

Considering that the measured signal S_m contains noise σ^2, which we assume is the same for all channels, we obtain the following relations to the “true” signal S_t for each coil:

$$S_{m,i}^2 = S_{t,i}^2 + \sigma^2$$

For the combined sum of squares signal this yields:

$$S_m^2 = 3S_{m,1}^2 + 3S_{m,2}^2 = 3S_{t,1}^2 + 3S_{t,2}^2 + 6\sigma^2 = S_t^2 + 6\sigma^2$$

The best guess for the noise-corrected signal S_c is therefore $S_c = \sqrt{S_m^2 - 6\sigma^2}$.

Data exclusion criterion (to avoid ADC underestimation by noise “pollution”)

Looking at individual coil channels, we find:

$$S_{c,1}^2 = S_{m,1}^2 - \sigma^2 = 4S_{m,2}^2 - \sigma^2 = 4\frac{S_m^2}{15} - \sigma^2$$

$$S_{c,2}^2 = S_{m,2}^2 - \sigma^2 = S_{m,2}^2 - \sigma^2 = \frac{S_m^2}{15} - \sigma^2$$

Our criterion for data exclusion was that the squared corrected signal in one of the lower coils $S_{c,2}^2$ is less than $3\sigma^2$. This yields $S_{c,2}^2 = \frac{S_m^2}{15} - \sigma^2 < 3\sigma^2$, which can be transformed into $S_m < \sqrt{60}\sigma$.

Supporting Information: Effective gradient profiles and water reference ADCs:

The following supporting figure contains the effective gradient profiles for monopolar **(a)** and flow-compensated diffusion weighting **(b)**, which were used for the Monte Carlo simulations. In addition, the ADC values measured in a water reference tube simultaneously with the blood samples are displayed for monopolar **(c)** and flow-compensated gradients **(d)**. The same markers as for the blood samples shown in FIG.2 in the manuscript are used to allow one to identify the water samples that was measured together with a particular blood sample.

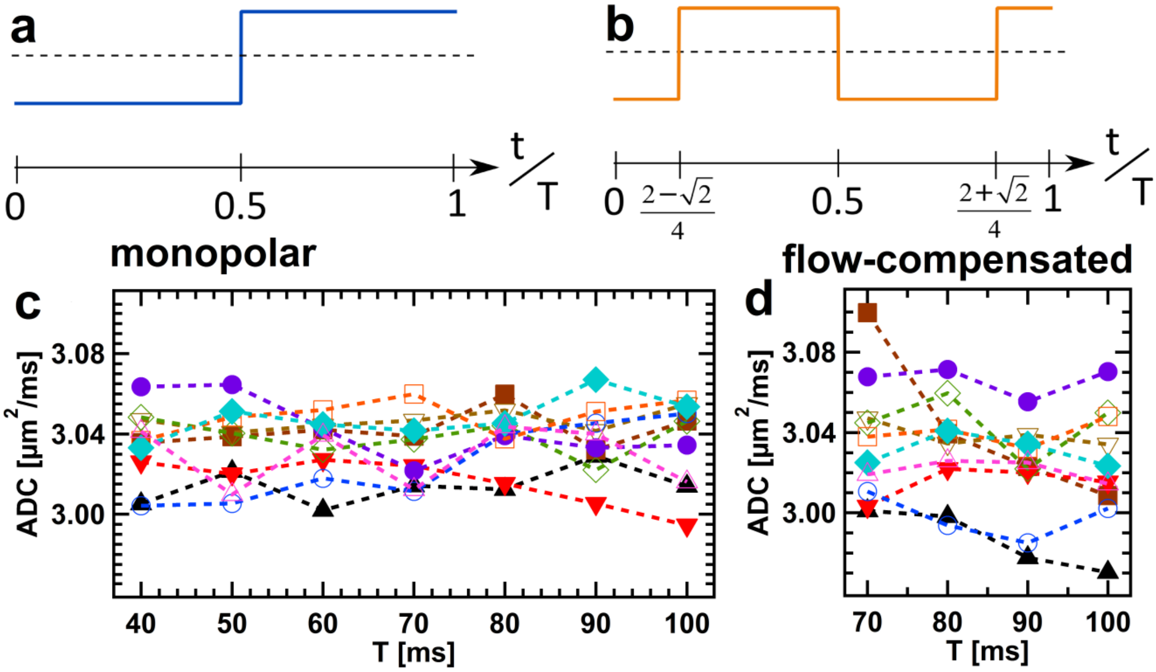

Supporting Fig. S1: (**a**,**b**): Effective diffusion weighting profiles used for the Monte Carlo simulations, which correspond to the diffusion sequence displayed in FIG.1b in the manuscript. To obtain a particular b-value, the waveforms have to be scaled by the gradient amplitude g such that $b = a\gamma^2 g^2 T^3$ with $a = 1/12$ for the monopolar profile **(a)** and $a = \sqrt{2}/\,8 - 1/6$ in the flow-compensated case **(b)**. (**c**,**d**): ADCs measured in a water reference tube simultaneously with the blood samples (same marker as the respective samples in FIG. 2). Water ADCs agree within 3% error across measurements and with literature values.
